# Derechos sexuales y reproductivos para la anticoncepción en Bolivia, Colombia y Uruguay en el marco de los derechos humanos

**DOI:** 10.26633/RPSP.2017.140

**Published:** 2017-12-05

**Authors:** Kathya Lorena Cordova-Pozo, Graciela Cordova-Pozo, Ana Monza, Gabriela Píriz, Diva Moreno-Lopez, Ivan Cardenas

**Affiliations:** 1 South Group South Group Cochabamba Bolivia South Group, Cochabamba, Bolivia; 2 Hospital Seton Cochabamba Cochabamba Bolivia Hospital Seton, Cochabamba, Bolivia; 3 Administración de los Servicios de Salud del Estado (ASSE) Administración de los Servicios de Salud del Estado (ASSE) Montevideo Uruguay Administración de los Servicios de Salud del Estado (ASSE), Montevideo, Uruguay.; 4 Ministerio de Salud y Protección Social Ministerio de Salud y Protección Social Bogotá Colombia Ministerio de Salud y Protección Social, Bogotá, Colombia.

**Keywords:** Anticoncepción, legislación, legislación sanitaria, Bolivia, Colombia, Uruguay, adolescente, derechos humanos, Contraception, health legislation, Bolivia, Colombia, Uruguay, adolescent, human rights, Anticoncepção, legislação sanitaria, Bolívia, Colômbia, Uruguai, adolescente, direitos humanos

## Abstract

**Objetivo.:**

Realizar una comparación entre las Directrices de la Organización Mundial de la Salud (OMS) para la anticoncepción en el marco de los derechos humanos (DDHH) con el marco normativo existente en Bolivia, Colombia y Uruguay y evaluar los aspectos que son necesarios desarrollar en la normativa.

**Métodos.:**

Se realizó un análisis sistemático con base al marco analítico de la OMS ¨Respeto de los DDHH cuando se proporciona información y servicios de anticoncepción: orientación y recomendaciones¨ para determinar si la legislación de Bolivia, Colombia y Uruguay contienen referencias generales a la población, referencias específicas para los adolescentes o no hacen referencia.

Para este fin, se analizó un total de 36 documentos relacionados con la anticoncepción: 9 de Bolivia, 15 de Colombia y 12 de Uruguay.

**Resultados.:**

Se verificó que la legislación de cada país cumple con varias recomendaciones de la OMS. Los tres países tienen fortalezas en la no discriminación y el espacio para las decisiones informadas; sus debilidades están en la accesibilidad, la calidad y la rendición de cuentas. La aceptabilidad es una fortaleza para Colombia y Bolivia; la confidencialidad es para Bolivia y Uruguay. Colombia tiene como debilidad la disponibilidad, la confidencialidad y la participación.

**Conclusiones.:**

La comparación de la legislación nacional con la guía de la OMS ayuda a ver las fortalezas y las debilidades en el marco normativo nacional y ver oportunidades para mejorar la normativa.

## Abstract (en)

**Objective.:**

Compare World Health Organization (WHO) guidelines for contraception in a human rights framework with the existing regulatory frameworks of Bolivia, Colombia, and Uruguay and evaluate which aspects of those regulations need to be developed.

**Methods.:**

A systematic analysis was based on the WHO analytical framework “Ensuring human rights in the provision of contraceptive information and services: Guidance and recommendations” to determine whether the legislation of Bolivia, Colombia, and Uruguay contain general references to the population, specific references to adolescents, or do not refer to the topic. To this end, 36 documents related to contraception were analyzed: 9 from Bolivia, 15 from Colombia, and 12 from Uruguay.

**Results.:**

It was confirmed that each country’s legislation complies with several WHO recommendations. The three countries have strengths in nondiscrimination and in opportunity for informed decision-making, and have weaknesses in accessibility, quality, and accountability. Acceptability is a strength in Colombia and Bolivia, and confidentiality is a strength in Bolivia and Uruguay. Colombia has weaknesses in availability, confidentiality, and participation.

**Conclusions.:**

Comparison of national legislation with WHO guidance helps to see the strengths and weaknesses of national regulatory frameworks and to see opportunities to improve regulations.

## RESUMO

**Objetivo.:**

Comparar as diretrizes da Organização Mundial da Saúde (OMS) para contracepção como parte dos princípios dos direitos humanos com os enquadramentos regulamentares existentes na Bolívia, Colômbia e Uruguai e avaliar os elementos destes enquadramentos que precisam ser melhorados.

**Métodos.:**

Realizou-se uma análise sistemática segundo a metodologia analítica descrita no documento da OMS ¨Respeito aos direitos humanos ao prestar informações e serviços sobre contracepção: orientação e recomendações com o propósito de verificar se as legislações da Bolívia, Colômbia e Uruguai fazem referências gerais à população, referências específicas aos adolescentes ou não fazem referências. Ao todo, 36 documentos sobre contracepção foram analisados: 9 provenientes da Bolívia, 15 da Colômbia e 12 do Uruguai.

**Resultados.:**

Verificou-se que as legislações dos três países cumprem com diversas recomendações da OMS. Não discriminação e oportunidade para decidir de forma esclarecida são os pontos fortes e acessibilidade, qualidade e prestação de contas são os pontos fracos. A aceitabilidade é um ponto forte na Colômbia e Bolívia e a confidencialidade, na Bolívia e Uruguai. Disponibilidade, confidencialidade e participação são os pontos fracos na Colômbia.

**Conclusão.:**

A comparação das legislações nacionais com o guia da OMS possibilita identificar os pontos fortes e fracos no enquadramento regulamentar nacional e encontrar oportunidades para melhorar.

La salud sexual y reproductiva (SSR) es un tema crítico para los adolescentes (entre 11–19 años de edad) porque las acciones durante esta etapa pueden tener consecuencias para su vida, como la maternidad adolescente, infecciones de transmisión sexual (ITS) o la infección por el virus de inmunodeficiencia humana (VIH). La población adolescente es 22% en Bolivia, 18,4% en Colombia y 15,3% en Uruguay. Bolivia y Colombia tienen 13% de mujeres adolescentes casadas, y 20% de 18 años o menos con embarazo (1). En Uruguay, el número de nacimientos en madres adolescentes es de 16,4%; mientras 3,6% son madres en un entorno económico favorable, un 22,4% es madre en uno desfavorable (2).

Con respecto al VIH, el 31,5% de la población en estudio en Bolivia y 26% en Colombia conoce sobre esta entidad nosológica, en el área urbana, 8,9% y 17,6% en área rural respectivamente; la prevalencia del VIH en la población de 15-24 años es 0,3% en Bolivia y 0,5 en Colombia y Uruguay (1).

La prevalencia de métodos anticonceptivos (MAC) modernos, es 33,7% en Bolivia, 72,7% en Colombia y 74,8% en Uruguay; siendo el condón el método menos usado: 4% en Bolivia, 7% en Colombia, y 30,8% en Uruguay (1). Esta realidad se repite en Sudamérica con contrastes entre regiones, área rural-urbana, y contextos socioeconómicos, debido a que Sudamérica tiene altos niveles de desigualdad que obstaculizan la inclusión social y un crecimiento sostenible (3).

El Consenso de Montevideo insta a los gobiernos a cambiar sus legislaciones con el fin de garantizar el ejercicio de los derechos sexuales y reproductivos (DSyR) (4). Este Consenso es un marco estratégico de avance del Programa de Acción de la Conferencia Internacional sobre Población y Desarrollo de El Cairo en 1994 y el Consenso Latinoamericano y del Caribe sobre Población y Desarrollo, aprobado en México en 1993.

El objetivo de este manuscrito es realizar una comparación entre las recomendaciones de la OMS (5) para proporcionar información y servicios de anticoncepción con el marco normativo de Bolivia, Colombia y Uruguay, evaluando la legislación sobre anticoncepción y resaltando los aspectos necesarios para desarrollar y lograr el respeto de los DDHH.

## MÉTODOS

Se realizó un análisis y evaluación sistemática basada en el marco analítico, circunscrito en el documento de la OMS ¨Respeto de los derechos humanos cuando se proporciona información y servicios de anticoncepción: orientación y recomendaciones¨ (2014) (cuadro 1). Se evaluó si la legislación de Bolivia, Colombia y Uruguay contienen referencias generales a la población, referencias específicas para adolescentes, o no se hace referencia a estas recomendaciones. Estudios similares ya fueron realizados bajo esta metodología (6,7). El análisis sólo presenta la legislación existente y no su grado de implementación.

Se coleccionaron y revisaron 36 documentos incluyendo leyes, decretos, resoluciones, políticas, guías y manuales relacionados con anticoncepción, 9 de Bolivia, 15 de Colombia y 12 de Uruguay (anexo 1). El análisis fue multidisciplinario y por país; intervinieron, por Bolivia una economista política y una médica del sistema público; por Colombia una líder técnica del adolescente y un profesional especializado del Ministerio de Salud y Protección Social; y por Uruguay una psicóloga y una médica ginecóloga del área de SSR del sistema público.

## RESULTADOS

Los resultados se presentan en el orden numérico bajo el marco analítico de la OMS denominado “Respeto de los derechos humanos cuando se proporcionan información y servicios de anticoncepción: orientación y recomendaciones” (OMS, 2014) (cuadro 1)^a^

### 1) Discriminación en la provisión de anticonceptivos información y servicios [1.1-1.2]

Los tres países tienen normativa para garantizar el acceso a la información y servicios de anticoncepción sin discriminación [1.1], Bolivia se enfoca en que todo adolescente saludable sea elegible para cualquier MAC previa orientación y asesoramiento; y concebir establecimientos amigables que atiendan particularidades regionales y culturales para favorecer el acceso y uso de anticonceptivos por múltiples usuarios, entre ellos adolescentes (8,9). Colombia garantiza los DSyR libres de violencia, en igualdad, libertad, autonomía y sin discriminación por sexo, edad, etnia, orientación sexual, identidad de género, discapacidad, religión o víctima de conflicto armado (10,11); detectar y atender los factores de riesgo, promoviendo factores de protección para adolescentes (12). Uruguay incluye la universalización del nivel primario de atención en SSR con integralidad, calidad, oportunidad, compromiso de los recursos humanos y sistemas de información adecuados, garantizando el acceso universal a MAC (13,14).

Los tres países respaldan con leyes y políticas a los programas establecidos [1.2]. Bolivia, se enfoca en la no discriminación (15) y se basa en principios básicos del derecho a la vida como la integridad física, la equidad de género y generacional, participación, solidaridad, justicia social y reciprocidad, con respeto a la diversidad cultural (16); se promueve la sensibilización para los adolescentes y la capacitación en servicios diferenciados (17). La política de sexualidad de Colombia se articula con las normas nacionales e internacionales sobre sexualidad y DSyR (11), lo que ayuda a lograr objetivos comunes como la Atención Primaria en Salud, el Plan Decenal de Salud Pública 2012-2021, el Sistema General de Seguridad Social en Salud (SGSSS, Ley 100/93) que reglamenta el Plan de Atención Básica^1^ y los programas de prevención de ITS/VIH y embarazos no deseados a través de información, educación, comunicación y uso del condón masculinois, garantizando el acceso gratuito de adolescentes a los servicios de anticoncepción (18-21). Uruguay se enfoca en protocolizar la atención para anticonceptivos e infertilidad para toda la población (13).

### 2) Disponibilidad de anticonceptivos información y servicios [2.1]

Bolivia da criterios de programación y almacenamiento anual basados en una meta programática para cada establecimiento de salud y se agrega a nivel municipal, local, departamental y nacional (8) para fortalecer el Sistema Nacional Único de Suministro y el Subsistema de Administración Logística de Medicamentos e Insumos, que aseguren la provisión de materiales, medicamentos y MAC (16). No se encontró normativa específica para Colombia; Uruguay tiene normativa para compras que revisa, consolida y estima las necesidades anualmente, con base al consumo de insumos y medicamentos de prestadores de servicios de salud coordinado por el área de SSR, asegurando el acceso con calidad (22, 23).

**CUADRO 1.**
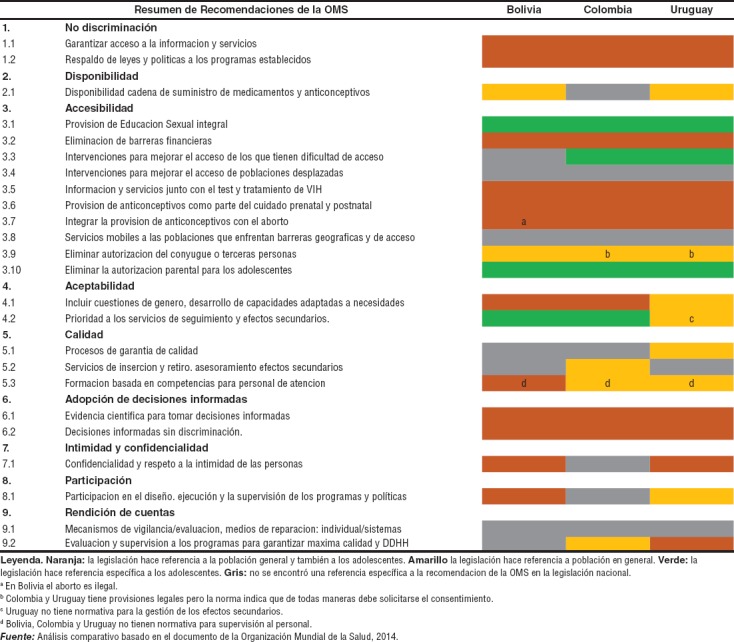
Análisis comparativo de la Legislación en Bolivia, Colombia y Uruguay bajo el marco analítico de la OMS “Respeto de los derechos humanos cuando se proporcionan información y servicios de anticoncepción: orientación y recomendaciones” (OMS, 2014)

### 3) Accesibilidad de los anticonceptivos información y servicios [3.1-3.10]

Los tres países cumplen con 4/10 sub-recomendaciones y falta mejorar el acceso de poblaciones desplazadas y las que viven en situación de crisis [3.4]; y servicios móviles para los que enfrentan barreras geográficas [3.8].

Los tres países tienen normativa para educación integral en sexualidad (EIS), indicando que las instituciones educativas y centros de salud están obligados a la educación, orientación en SSR, ejercicio responsable y libre de DSyR [3.1]. Para Bolivia, la EIS debe estar acorde al desarrollo físico y psicológico (17), la familia también debe educar (16). Colombia promueve el autoconocimiento, autoestima, identidad sexual con equidad, respeto, preparación para una vida familiar armónica y responsable de acuerdo a necesidades psíquicas, físicas, afectivas según su edad (24,25) ; donde la familia y escuela están obligadas a la formación, orientación y estimulación del ejercicio de derechos, responsabilidades y autonomía para un ejercicio responsable de DSyR y vida en pareja; y el sistema de seguridad social oferta servicios especializados para adolescentes (25). Uruguay se enfoca en la capacitación de docentes en escuelas, colegios y servicios de SSR para la orientación y prevención de ITS en todos los grupos etarios (13,14) y evitar situaciones de embarazo no deseado, enseñando “cómo negociar” el uso del condón (26,27).

Los tres países garantizan el acceso gratuito, seguro y confiable a información y servicios de anticoncepción para la población general y adolescentes [3.2]. Estos servicios incluyen dispositivo intrauterino (DIU), anticoncepción oral (incluida la píldora de emergencia, en Bolivia solo está disponible en centros pilotos) y condón; aún no están disponibles los implantes de manera universal (8,13). En Bolivia, la anticoncepción es gratuita, es parte de los programas de planificación familiar y VIH/SIDA/ITS (16), evitando que el costo del servicio y los MAC limiten las opciones disponibles para los adolescentes (28). Colombia, apunta a la prevención de ITS/VIH y embarazos no deseados con distribución gratuita de métodos temporales a la población en establecimientos de salud, en el sistema de seguridad social y donde se realizan actos sexuales (18-20,29).

Uruguay tiene acceso universal, equitativo, continuo, de calidad a MAC reversibles seguros y confiables, los MAC irreversibles se reservan a mayores de 18 años o menores casadas, divorciadas o viudas 13-14); en el privado se entrega el condón por doce meses por una suma simbólica (26). El adolescente accede al método y a una consulta de promoción y prevención en SSR, distribución por máquinas expendedoras (26) y condón femenino (27,30).

Ningún país tiene normativa para mejorar el acceso a las zonas rurales y pobres de las ciudades [3.3]. Colombia y Uruguay dan información y acceso a adolescentes sobre MAC y uso del condón como parte de la población vulnerable (20,26). Colombia se enfoca en mejorar la salud de grupos de vulnerabilidad, de especial protección y de escasos recursos (31).

Ninguno de los países estudiados tiene normativa para mejorar el acceso de los desplazados o en situación de crisis [3.4]. Los tres países tienen normativa para el acceso universal a la prevención y tratamiento integral de ITS, VIH/SIDA, proporcionando información, consejería y entrega de condones con el test y tratamiento para población general y adolescentes [3.5] (8,18,19,26,27). Bolivia promueve el condón para evitar la transmisión (8). Colombia se enfoca en consejería y tratamiento a embarazadas (21). Uruguay contextualiza al adolescente, género y sus prácticas sexuales (27).

Los tres países tienen normativa para anticoncepción para prevenir un embarazo no deseado y postnatal [3.6] (8,20,26,32,33). Uruguay se enfoca en orientación y escucha a adolescentes, informa sobre el uso correcto del condón y otras recomendaciones para prevenir la reiteración de embarazos no deseados (26).

Los tres países tienen normativa para provisión de anticonceptivos postaborto [3.7] (8,20,34). En Bolivia, el aborto es ilegal. Colombia permite consentir un aborto (también al adolescente) (35).

En Uruguay el aborto voluntario esta despenalizado y se realiza asesoramiento en anticoncepción post evento proporcionando el MAC elegido por la mujer o la adolescente (34).

Ninguno de los países posee normativa sobre servicios móviles para reducir barreras geográficas [3.8].

Con respecto a la eliminación de autorización del cónyuge o terceros [3.9]: en Bolivia, cualquier mujer y hombre puede someterse a esterilización de larga duración o permanente de manera segura previo asesoramiento y consentimiento informado, sin consentimiento de la pareja (8); se estimula la corresponsabilidad en adolescentes, pero si asiste solo, se debe dar la información necesaria y puede elegir el método deseado, aunque no se consensue con su pareja (28). Colombia y Uruguay enfatizan la libre decisión y autonomía, aunque refieren que es práctica común pedir la autorización de la pareja por precaución en cirugías de esterilización femenina, no así para la masculina (11,34).

Los tres países tienen normativa para no requerir el consentimiento de los padres, para el adolescente, la edad no es razón médica para negar consejería en SSR o acceder a un método anticonceptivo [3.10] (21,26,28,36). Los tres ofertan métodos temporales con solo la solicitud y libre consentimiento. Bolivia presta especial cuidado con la oferta de métodos permanentes (28). Colombia además garantiza el acceso a información y educación (20,37). Uruguay oferta algunos tratamientos con concurrencia de padres, respetando la autonomía progresiva (13,38).

### 4) Aceptabilidad de información y servicios de anticoncepción [4.1-4.2]

Los tres países poseen normativa para proveer información y servicios aceptables basados en necesidades, particularmente de adolescentes con enfoque de género [4.1] (12,13,28). Bolivia indica orientación y escucha a partir de las inquietudes del adolescente para utilizar un MAC, instruye favorecer corresponsabilidad, generar habilidades de negociación, informar sobre la eficacia y efectos secundarios, consultas de seguimiento, tomar en cuenta tanto a los casados como a los solteros (8,28). Para Colombia, los adolescentes son parte transversal de su política, se enfoca en género, determinantes sociales para erradicar violencias de género, sexual y prevención del VIH/SIDA, discriminación por orientación sexual o identidad de género (12). Uruguay se enfoca en la formación del personal sanitario para decisiones libres (13).

En relación a los servicios de seguimiento y efectos secundarios [4.2]: Bolivia cuenta con los siguientes servicios de rastreo de prioridad y efectos secundarios al adolescente: a) 8 semanas después y cada 3 meses para consulta de control sobre el uso del método, consistencia, experiencias y dificultades; y b) 12 meses después para examen pélvico, PAP, investigar infecciones genitales, y efectos secundarios para cada método (8,28), pero no hay actividades de seguimiento a la población. Colombia por su parte, se enfoca en un seguimiento al adolescente vulnerable para adherencia al método y asistencia a consultas de seguimiento (20). Uruguay establece consulta con ginecología tres meses luego de iniciado un método (39) pero no tiene normativa para la gestión de los efectos secundarios.

### 5) Calidad de la información y servicios de anticoncepción [5.1-5.3]

Bolivia y Colombia no cuentan con normativa en procesos de garantía de calidad [5.1]. En Uruguay, el Ministerio de Salud define contenidos, planifica actividades de sensibilización y capacitación a profesionales de referencia para mejorar la calidad de atención (13).

En el caso de Bolivia y Uruguay no muestran normativa de servicios de inserción y retiro del DIU o implantes [5.2] y tampoco de asesoramiento sobre efectos secundarios. Colombia oferta la aplicación y retiro del DIU e implante de anticonceptivo subdérmico y se asesora sobre efectos secundarios (29).

Los tres países tienen personal de atención con formación basada en competencias [5.3]. Bolivia se enfoca en brindar orientación y atención con personal calificado para la comprensión, manejo de políticas y normas vigentes en anticoncepción y su aplicación en la promoción de los DSyR y acceso a orientación y servicios (8) para adolescentes. El personal debe tener destrezas en manejo de adolescentes, familias, comunidad y habilidades comunicacionales para garantizar la atención integral, ser imparcial sin emitir juicios de valor (28). Colombia indica que los profesionales de medicina o enfermería deben estar capacitados en inserción, retiro del DIU, consejería y obtener consentimiento informado (29). Uruguay se enfoca en la formación adecuada de aspectos técnicos, información, habilidades para comunicación y trato; prestaciones de salud para prevenir violencia física, psicológica, sexual, conductas discriminatorias (13), capacitación en orientación, escucha, y entrega de preservativo (26). Bolivia, Colombia y Uruguay no tienen normativa para supervisión al personal.

### 6) Adopción de decisiones informadas [6.1-6.2]

Los tres países tienen normativa que promueve la información y asesoramiento sobre MAC con el fin de que los usuarios puedan realizar su propia elección informada, dando libertad al adolescente de elegir después de recibir información sobre los MAC disponibles, ventajas, desventajas, riesgos, consecuencias de uso y otros, para que elija con base a sus necesidades y de forma integral [6.1] (8,11,30). Bolivia enfoca la normativa a la prevención de embarazos no deseados, al espaciamiento de hijos (16); y al ser integral para adolescentes, (9). Colombia valora la decisión personal como máxima expresión de libertad individual y ciudadana en los contextos laicos con conocimiento, razón, discernimiento, voluntad, asunción de límites y consecuencias de la decisión (11); para el adolescente se enfoca a la orientación para uso sistemático del método elegido (20). Uruguay se enfoca a orientar con base al ciclo vital, estilo de vida, valores, patrón de actividad sexual y aceptabilidad del método, resaltando la seguridad, eficacia, comodidad y accesibilidad (30); basada en género, derechos y diversidad para el ejercicio de una sexualidad placentera, libre y responsable (26).

Los tres países tienen normativa para la toma de decisiones libres, para el uso de MAC en forma correcta, tolerancia y mejora en su calidad de vida tanto para adolescentes y población en general [6.2]. Bolivia, se enfoca en la adecuación intercultural y respeto a la autodeterminación de las mujeres, donde, quien interviene para facilitar el ejercicio de derechos sobre el uso de un MAC; reconozca y respete su capacidad para tomar decisiones (8,16). Todo adolescente puede consultar por anticoncepción; los servicios de salud deben respetar ese derecho y ayudar a evaluar la decisión (profesional, personal o de pareja) (28). Para Colombia, el consentimiento informado, significa que las personas puedan decir que le fueron explicados y saben de MAC temporales y de largo plazo, los efectos, y pueden elegir libremente o cambiar su decisión antes del procedimiento sin riesgo; los adolescentes pueden solicitar libremente y consentir cualquier MAC no definitivo (20). Uruguay deja la elección del método al adolescente y el profesional asesora y acompaña este proceso explicando los motivos que apoyan o desaconsejan el uso del método basado en evidencia (26).

### 7) Intimidad y confidencialidad [7.1]

Bolivia y Uruguay tienen normativa para regular y respetar la intimidad y confidencialidad (13,16,31,40). Bolivia se enfoca en adolescentes (28), Uruguay en garantizar la calidad y privacidad de las personas (13). Colombia tiene confidencialidad y privacidad en general, no explícito para MAC ni adolescentes (31).

### 8) Participación [8.1]

En Bolivia, existen normas para participación en salud y SSR con consejos locales de juventud y grupos de control social para garantizar la calidad de los servicios (31). La norma provee la capacitación a jóvenes para ejercer sus derechos, toma de decisiones, autogestión de su salud y proyectos de vida.

La norma de anticoncepción promueve la participación de la comunidad en la promoción de los DSyR y actividades intersectoriales, indicando que la participación y corresponsabilidad es compartida entre el Estado, la sociedad y los jóvenes para la creación, ejecución y control de políticas de transformación social, política, económica y cultural (31). Colombia tiene una normativa para organizar una Comisión Nacional intersectorial para promocionar y garantizar DSyR conformada por unidades de gobierno sin incluir adolescentes ni población en general (41). Uruguay promueve la coordinación interinstitucional con participación de redes sociales y usuarios para intercambiar información, educación para la salud y apoyo solidario y participación activa en la implementación y monitoreo de acciones en SSR (13,42).

### 9) Rendición de cuentas [9.1-9.2]

Ningún país tiene mecanismos de responsabilización con respecto a la información y los servicios de anticoncepción proporcionados, que incluyan medios de compensación al individuo.

Los tres países poseen sistemas de evaluación y supervisión para garantizar la calidad, pero, solo Colombia tiene un sistema de evaluación y supervisión para garantizar los DDHH. En Bolivia, Colombia y Uruguay se encontró normativa para fortalecer los modelos de gestión, seguimiento, evaluación, control de implementación, y logro de los objetivos de la política mediante la recolección de datos a través de Comités de Análisis de la Información en Bolivia (16); Sistema de Información de la Protección Social en Colombia; y la incidencia y mecanismos de transmisión del VIH/SIDA/ITS en Uruguay (13).

Bolivia se enfoca en el conocimiento para reasignación de presupuestos y logro de metas (17). Colombia se enfoca en garantizar el respeto a la libertad de pensamiento, libre expresión en sexualidad y reproducción, minimizando el juzgamiento en procesos de atención en salud por posturas políticas, religiosas o culturales (11). Uruguay tiene supervisión a instituciones prestadoras de SSR con Equipos Coordinadores de Referencia para garantizar prestaciones establecidas en la Ley 18.426 con monitoreo y evaluación, identificación de barreras, de facilitadores, coordinación y usuarios para monitorear las prestaciones y elaborar mecanismos de evaluación (43).

## DISCUSIÓN

Este estudio verificó que las legislaciones de los tres países cumplen con varias recomendaciones de la OMS para información y servicios de anticoncepción en el marco de los derechos humanos (5). Los tres países tienen fortalezas en la no discriminación [1] y espacios para adopción de decisiones informadas [6]. Sus debilidades están en accesibilidad [3], calidad [5] y la rendición de cuentas [9]. La aceptabilidad [4] es una fortaleza para Colombia y Bolivia; la confidencialidad [7] es para Bolivia y Uruguay. Colombia tiene como debilidad la disponibilidad [2], la confidencialidad [7] y participación [8] (cuadro 1).

Los desafíos de estos países residen en reformar las leyes para optimar la provisión en anticoncepción en un marco de DDHH y hacer los MAC inclusivos a la población y los adolescentes (cuadro 2). La mejora a la legislación debe abordar un entorno articulado basado en debilidades y fortalezas del contexto nacional y del sistema de salud, educación, economía y comunidad para que se pueda implementar efectiva y equitativamente (3).

**CUADRO 2.** Oportunidades para fortalecer la legislación en Bolivia, Colombia y Uruguay

**Disponibilidad [2], accesibilidad [3] de información y servicios para los MAC:**
- Col: Integrar los MAC en la cadena de medicamentos esenciales para su disponibilidad [2.1]
- Bol: Crear intervenciones para el área rural, zonas urbanas de bajos recursos [3.3]
- Bol/Col/Uru: Incluir intervenciones para acercar a los desplazados y en crisis [3.4]
- Bol/Col/Uru: Incluir servicios móviles para reducir barreras geográficas [3.8]
- Col/Uru: Explicitar mecanismos de supervisión para no requerir la autorización del cónyugue [3.9]
**Acceptabilidad [4], calidad [5], confidencialidad [7] de información y servicios para los MAC:**
- Uru: Agregar normativa para la gestión de los efectos secundarios [4.2]
- Bol/Col: Integrar procesos de garantía de calidad al programa de anticoncepción [5.1]
- Bol/Uru: Incluir explicitamente los MAC reversibles de acción prolongada, la inserción y retiro [5.2]
- Bol/Col/Uru: Incluir normativa específica para supervisión al personal [5.3]
- Col: Incluir normativa específica para respetar la intimidad de las personas [7]
**Participación [8] y responsabilidad [9] de información y servicios para los MAC:**
- Col: Incluir normativa específica para la participación de personas en el diseño, ejecución y supervisión [8]
- Bol/Col/Uru: Incluir mecanismos de monitoreo y evaluación para medios de reparación [9.1]
- Bol: Introducir mecanismos de monitoreo para la protección de los derechos humanos [9.2]

Bol. Bolivia; Col .Colombia; y Uru. Uruguay.

Los numeros en corchetes hacen referencia a los item del cuadro 1.

***Fuente:***Elaboracion propia con base al documento de la Organización Mundial de la Salud (2014) y la revisión de la normativa.

Esta revisión se construye sobre análisis similares en Paraguay y Sudáfrica (6,7). Junto con Paraguay, Bolivia, Colombia y Uruguay reconocen que los adolescentes tienen necesidades especiales y requieren legislación específica en anticoncepción bajo la perspectiva de DDHH de la cual también la población se benefidesafýoscia. Los cuatro comparten similares desa-fíos en accesibilidad [3], calidad [5], participación [8] y rendición de cuentas [9] y al ser firmantes del Consenso de Montevideo podrían trabajar en consensos multinacionales para mejorar estas áreas.

Una limitación de este estudio es que analiza el marco normativo y no su implementación. Acciones realizadas para mejorar los servicios e información de anticoncepción, así como los retos que enfrenta cada país por su sistema de salud, situación socioeconómica, tabús, grado de implementación de leyes y no reconocimiento de los derechos pueden hacer que un país cumpla más rápido o no las recomendaciones de la OMS. Futuros estudios podrían realizar el análisis de la implementación y las limitantes para una visión integral sobre anticoncepción.

Se concluye que la comparación de la legislación nacional de Colombia, Bolivia y Uruguay con la guía de la OMS para anticoncepción visualiza fortalezas y debilidades en el marco normativo nacional y regional para encontrar oportunidades de robustecer la normativa. Un marco normativo que garantiza respeto a DDHH avala el compromiso político en materia de desarrollo promueve las buenas prácticas hacia un servicio de calidad.

## Declaración.

Las opiniones expresadas en este manuscrito son responsabilidad del autor y no reflejan necesariamente los criterios ni la política de la *RPSP/PAJPH* y/o de la OPS

## Footnotes

a En este manuscrito los números entre corchetes hacen referencia al ítem enumerados en el cuadro 1.

1 Este programa enmarca tanto el Plan de Intervenciones Colectivas (PIC) como el Plan Obligatorio de Salud (POS)
